# Payment Reforms for Prehospital Care Services in a Middle Income Country: Assessing Implementation and Patient Outcomes Using a Mixed-Methods Approach

**DOI:** 10.1155/2018/9298024

**Published:** 2018-07-09

**Authors:** Paibul Suriyawongpaisal, Samrit Srithamrongsawad, Pongsakorn Atiksawedparit, Khanisthar Phooseemungkun, Krongkan Bunchaiyai, Thanita Thongtan

**Affiliations:** ^1^Department of Community Medicine, Faculty of Medicine, Ramathibodi Hospital, Mahidol University, Rama 6, Ratchatewi, Bangkok, Thailand; ^2^Department of Emergency Medicine, Faculty of Medicine, Ramathibodi Hospital, Mahidol University, Rama 6, Ratchatewi, Bangkok, Thailand; ^3^National Institute of Emergency Medicine, Ministry of Public Health, Tiwanond, Muang, Nonthaburi, Thailand; ^4^Department of Physiology, Faculty of Science, Mahidol University, 272 Rama 6 Rd, Rajathevi, Bangkok 10400, Thailand

## Abstract

**Introduction:**

Financing health systems constitutes a key element of well-functioning healthcare system. Prior to 2015, two new financial arrangements (direct-pay and E-claim systems) were introduced on a voluntary basis which aimed to pool more financial resources and improve cash flow of prehospital care systems. The aims of this study were to (1) assess the effects of direct-pay system in terms of (a) timeliness of reimbursement to EMS agencies, (b) changes in clinical care processes, and (c) the outcomes of patient care as compared to previous system; (2) identify the reasons for or against EMS agencies to participate in direct-pay system mechanisms; (3) identify the emerging issues with potential to significantly further the advancement of EMS systems. Using a mixed-methods approach, retrospective datasets of 3,769,399 individual records of call responses from 2015 to 2017 were analyzed which compared EMS units with the direct-pay system against those without in terms of time flow of claim data and patient outcomes. For qualitative data, in-depth interviews were conducted.

**Results:**

EMS units participating in both systems had the highest percentages of financial claim being made in time as compared to those not participating in any (p=0.012). However, there were not any practically meaningful differences between EMS units participating and not participating in either of the payment systems in terms of patient care such as appropriateness of response time, airway management, and outcome of treatment. Analysis of data from focus-group and individual interviews ended up with a causal loop diagram demonstrating potential explanatory mechanisms for those findings.

**Conclusion:**

It is evident that progress has been made in terms of mobilising more financial inputs and improving financial information flow. However, there is no evidence of any changes in patient outcomes and quality of care. Furthermore, whether the progress is meaningful in filling the gaps of financial demands of the prehospital care systems is still questionable. Room for future improvement of prehospital care systems was discussed with implications for other countries.

## 1. Introduction

The development and implementation of an EMS system vary among lower-middle income countries (LMICs). Many LMICs lack an organized EMS system with most ambulances used purely for transport and not as emergency care vehicles. Financial issues are the most common problems faced by LMICs [1].

In effect, developed countries like the U.S. with the so-called modern EMS systems are still facing financial constraints. Since 2010, many changes enacted by the Patient Protection and Affordable Care Act (ACA) under the Obama Care Reform have directly affected emergency care with potential indirect effects on EMS systems. New Medicaid enrollees and changes to existing coverage plans may alter EMS transport volumes. Reimbursement changes such as adjustments to the ambulance inflation factor (AIF) alter the yearly increases in EMS reimbursement by incorporating the multifactor productivity value into yearly reimbursement adjustments [2].

Another example is payment to EMS agencies in New Zealand which was found to be inconsistent in terms of payment rate and beneficiary groups among funders (the Ministry of Health and the Accident Compensation Commission, (ACC)). Under the ACC payment systems focusing on transporting live injured patients, EMS providers were incentivised to overtriage practices and perceived pressure to transport patients. In addition, only ambulance services were considered eligible for the ACC payment system despite the fact that fire services increasingly attended to emergency cases as first responders [3].

**Figure 1:**
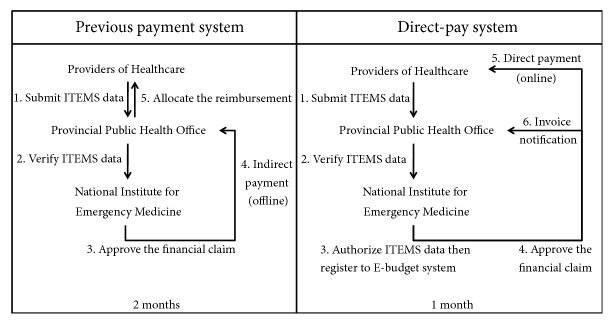
A comparison between the data flows of previous payment system and direct-pay system.

Thailand, a middle income country, formalised the development of EMS systems by establishing a national statutory lead agency, the National Institute for Emergency Medicine (NIEMS), in 2008. Since then NIEMS sought to focus its mission on strengthening EMS [4]. With expanded coverage of EMS and certain degree of performance improvement as indicated by shortened response time over the last decade, there is currently a growing concern of financial support, such as delayed payment to EMS agencies, provision of services without reimbursement, and overall deficit in the financial system. As a result, NIEMS took a policy of dubbed direct-pay system aiming at mobilisation of more financial resources and better facilitating the financial flows in 2016. The direct-pay systems channeled payment directly from the source to EMS units instead of indirect transfer through provincial health offices supervising EMS units in each province (Figure 1).

This study had two purposes. The first purpose was to assess whether progress has been made towards its aim. The second purpose was to draw lessons learnt to guide further improvement of the EMS systems. Specifically, we aimed to answer the following questions: (1) What are the effects of direct-pay system mechanisms in terms of timeliness of reimbursement to EMS agencies and changes in clinical care processes of patients as compared to previous mechanisms? (2) What are the reasons for or against EMS agencies to participate in direct-pay system mechanisms? (3) What are emerging issues with potential to significantly further the advancement of EMS systems after the introduction of the direct-pay system?

## 2. Methods

### 2.1. Study Design

Given the multiple objectives of the policy, we employed a mixed-methods approach comprising (a) secondary data analysis to provide quantitative assessment of the policy implementation (question no. 1) and (b) qualitative methods including documentary review, focus-group discussions, and informal individual interviews to address question no. 2 and no. 3.

### 2.2. Settings and Policy Interventions

Since 2009, NIEMS was established as a statutory national lead agency under the Ministry of Public Health [4]. It was mandated to formulate master plan and orchestrate its implementation for the development of EMS system including service standard setting, EMS personnel training and registering, financing of EMS services, and policy research. During 2009 to 2012, NIEMS chose to focus on expansion of infrastructure and mechanisms for prehospital care including manpower development and setting up new ambulance stations. This was built on the momentum of earlier efforts on prehospital care development which started at least 2 decades earlier. Prehospital ambulances were organized in 4 tiers (Advanced Life Support, Intermediate Life Support, Basic Life Support, and First Responder) with public financing system under NIEMS responsibility.

Under the EMS systems covering 68 million population in 76 provinces of the country, NIEMS has played a major role as a regulator and public purchaser of EMS services including operation of an emergency dispatch center for ambulances (EDC) in each province. In 2016, there were 8,669 registered EMS units under local authorities (64.7%), public hospitals (22.5%), voluntary organizations (9.7%), private hospitals (2.6%), and others (0.5%). They responded to 1.5 million calls in total per year [5].

Before the 2016 direct-pay system, NIEMS reimbursed EMS agencies by transferring the payment through provincial health office (PHO) in each province. The payment was made on fee-for-service basis with a fixed rate per call for each tier (i.e., $30 for Advanced Life Support, $15 for Basic Life Support, and $10.5 for First Responder). In 2015, NIEMS spent around $ 20 million on reimbursement of EMS operations [6]. The transfer of payment was made on monthly basis after receiving claims initially approved by the PHO, which usually took over 2 months for the whole process. At the start, each paper-based claim was filed to the PHO by EMS agencies upon completion of an operation using a standard form called “Information Technology for Emergency Medical System (ITEMS)”. The PHO would audit and approve the claim data before transferring (in bulk) to NIEMS. Then NIEMS would reaudit the claims before transferring the money to the PHO for further distribution to EMS units and EDCs. Under this payment system of paper-based financial transactions, keeping track of the claim amounts was almost impossible. In addition, an audit report of the reimbursement for EMS system in 2015 highlighted a concern of EMS agencies about provision of EMS services without being reimbursed [7]. As a result, NIEMS responded with the direct-pay system to channel the payment directly to EMS units and EDCs in 2016.

**Figure 2:**
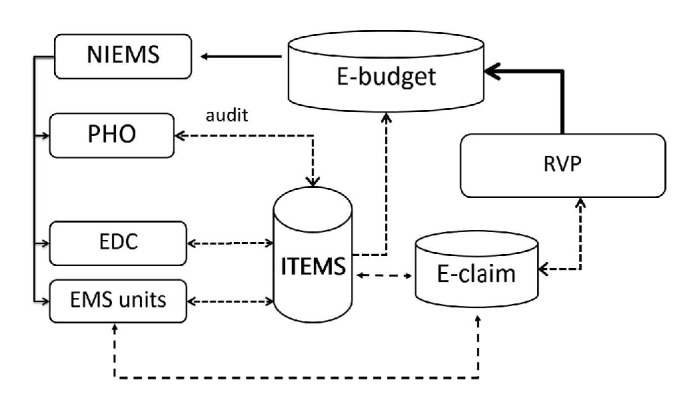
Data and financial flows under NIEMS and RVP collaboration on E-claim payment system. Note: EDC: emergency dispatch center; PHO: provincial health office; E-budget: electronic budgeting dataset under NIEMS (National Institute of Emergency Medicine); E-claim: electronic claim dataset under RVP (Road Accident Victims Protection Company Limited); dash line: data flows; solid line: financial flows.

To facilitate direct-pay system, NIEMS set up a web-based claim dataset (i.e., ITEMS dataset shown in Figure 2) allowing direct data entry by each EMS unit using a modified version of the standard form. However, the audit function remains at the PHO. Once the claims get final approval by NIEMS, the money is directly transferred to each EMS units' bank account. Figure 1 compared the data and financial flow between the previous payment system and the direct-pay system. Implementation of the direct-pay system has been undertaken on voluntary basis; i.e., each EMS unit had its own choice of either joining the system or remaining with the previous payment system. In practice, a province participating in the system does not necessarily mean every EMS agency is taking part. So far there are 47 participating provinces (61.8%). Consequently, those nonparticipating provinces mean all EMS agencies are not participating. This is due to the fact that NIEMS approached the PHO of each province in order to implement the system.

In parallel to the direct-pay system, there is another ongoing financial arrangement which involves NIEMS in collaboration with a private insurance agency acting as a national clearing house mechanism. This insurance agency is responsible for the coverage of compensation to road-traffic-injury victims and EMS agencies (for ambulance services). Figure 2 shows data and financial flows under NIEMS and RVP collaboration on E-claim payment system. Data exchange takes place between ITEMS dataset and E-claim dataset with a major focus on avoiding duplicate payments. This could be achieved by making use of citizen identification number of road accident victims available on each of the datasets. It should be noted that ITEMS dataset handles data pertinent to all types of patients being transported by EMS units whereas E-claim dataset handles data specifically related to road accident victims regardless of transport status.

The insurance agency, Road Accident Victims Protection Company Limited (RVP), was set up in February 2003 as a result of revision of the Road Accident Compensation Act 1992 in order to facilitate more access of road-traffic-injury victims to compensation for loss. According to insurance statistics, loss ratios varied from 25% to 35% during 1997–2014 as a result of their compliance to the law [8]. A loss ratio is the ratio between total spending and total premiums collected. This indicated substantial deficit in coverage of the compensation for victims. In 2012, an online electronic claim system (dubbed E-claim system) was set up to better facilitate hospitals and EMS agencies in making claims. In principle, E-claim system intended to facilitate access to data elements required by the claim protocols such as registered number of vehicles involved in crash, victims' ID, motor-vehicle-insurance-policy number, and police records of a crash event. In 2015, NIEMS linked its electronic claim database (ITEMS) to the E-claim system of the company. Again, hospitals and EMS agencies are free to choose whether to use the facilities or not. It was expected that E-claim system would shorten the time of cash flow into hospitals or EMS agencies as well as improve coverage of the compensation to them and the victims (as they received medical care). Up to 2015, EMS agencies in 32 provinces joined the E-claim system (42.1%), and 19 of them overlapped with those joining the direct-pay system mentioned above.

### 2.3. Quantitative Methods

ITEMS datasets from 2015–2017 were eligible for the study. The dataset included 3,769,399 individual records of call responses from 76 provinces. This study examined the effect of both the direct-pay system and the E-claim system to the reimbursement data flows including time frame from call taking to complete submission of financial claims and percentages of financial claim being made in time. We also assessed the effect of those systems on the quality of care in terms of the appropriateness of response time, airway management, bleeding control, fluid replacement, fracture stabilization, and outcome of treatment. Normally, nurses at emergency rooms would audit these prehospital treatment practices and give feedback to EMS units on a regular basis with varying frequencies across provinces. This is a result of nation-wide clinical audit systems established by NIEMS since 2011 [9].

Categorical data were reported as number with its percentage. Continuous data were reported as mean (standard deviation; SD) or median (interquartile range; IQR) for normal and nonnormal distributions, respectively. For implementation outcome, multilevel mixed-effects linear regression was applied for comparing between participating and nonparticipating EMS units. In the absence of multicollinearity between direct-pay and E-claim systems, multivariate analysis by controlling year and system was applied. For patient outcomes, only patients who needed prehospital treatment were included for final analysis. Comparing fatal outcomes across EMS units with different participation in the initiatives, the whole dataset was included. Chi-square test was applied to compare patient outcomes between participating and nonparticipating EMS units. STATA version 14 (StataCorp 2015, Stata Statistical Software: Release 14, College Station, TX: StataCorp LP) was applied for all statistical analyses and p value less than 0.05 was considered statistically significant.

### 2.4. Qualitative Methods

Documentary review aimed to guide the interviews and triangulate findings from the interviews. Documents were searched purposively using Google Scholar and Google Search based on the following keywords: EMS, prehospital care, payment methods, reimbursement, access, staff mix, human resource, NIEMS, RVP, road victims, compensation, policy, annual report. Key actors mentioned above were contacted for relevant documents not accessible on the Internet.

Focus-group or informal individual interviews were done using the semi-structured interview guideline according to the WHO health systems building blocks [10] (Table 2). The interviewees at provincial level were purposively chosen under two categories: 4 provinces with participating EMS agencies in direct-pay system and 4 provinces without participating EMS agencies (Table 1). Tape records were transcribed verbatim. Thematic content analysis was performed to capture emerging themes, subthemes, and details. Relationships between themes and subthemes were identified. These were carried out in parallel by the first three authors. The authors discussed the findings to achieve consensus on the final conclusions. These qualitative methods would enable us to make better sense of the results from quantitative analysis of ITEMS dataset and comprehensive understanding of how the payment initiative might influence overall performance of the EMS systems.

## 3. Results

### 3.1. Effect of Direct-Pay System and E-Claim System on Reimbursement Data Flows

Table 3 revealed the time frame for data flows from call taking to completed submission of financial claim in the clusters of EMS units joining direct-pay system (n = 47 provinces) which were 10.47 days shorter than that in those clusters that did not join (n=29 provinces) (p=0.294). Similar effects were observed for the E-claim system (n=32 provinces) which were 8.07 days shorter than that in the previous system (n=44 provinces) (p=0.411). Categorical approach to the data confirmed the above findings for the direct-pay system (p=0.01) but not the E-claim system (p=0.181). There were no statistically significant increases of the percentages in the E-claim system EMS unit (coefficient = 9.69%, p=0.181). In Table 4, the EMS units participating in both systems had the highest percentages of financial claim being made in time as compared to those not participating in any (p=0.012).

Interviews' findings corroborated with the findings from quantitative analysis (Table 5). Almost all EMS practitioners and staff of PHO in provinces with participating EMS units believed that direct-pay system lessened the burden of work related to submission of financial claim as compared to the previous system based on paper work. Apart from facilitating claim data entry, direct-pay system also allows keeping track of the financial claims. Nonetheless, there were complaints about missing data due to unregistered motor vehicles or registered motor vehicles without insurance. The missing data rendered ineligibility to make claim as required by the RVP protocol.

### 3.2. Effect of Direct-Pay and E-Claim System on Delivery of Prehospital Care

Table 6 reveals a failure to detect any practically meaningful differences between EMS units participating and not participating in either of the payment systems in terms of the appropriateness of response time, airway management, bleeding control, fluid replacement, fracture stabilization, and outcome of treatment.

Similarly, reviewing annual reports of NIEMS from 2014 to 2016 showed slightly declining trends of performance of EMS units according to standards set by NIEMS. For instance, percentages of those meeting standard response times for call taking to on-scene arrival within 10 minutes slightly declined from 76 to 74 percent whereas the time frame for traveling from scene to hospital within 60 minutes declined from 99 to 97 percent. Again, looking into insurance statistics for potential effect of E-claim system on delivery of prehospital care revealed negative impact. According to insurance statistics, loss ratio has followed a declining trend for compensating road-traffic-injury victims related to crash involved in either 4 or more wheeled motor vehicles and for two wheeled motor vehicles during the last decade. This might suggest road accident victims who needed medical care had been increasingly less likely to access the care they needed.

**Figure 3:**
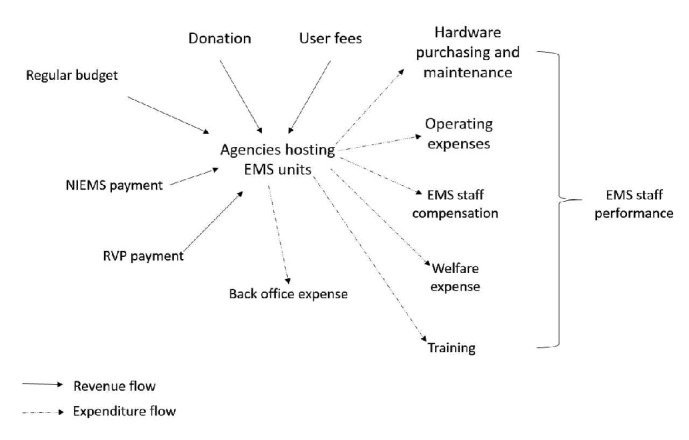
Causal loop diagram of potential explanatory mechanisms of EMS overall performance.

*Why Did Direct-Pay and E-Claim System Fail to Elicit Intended Effects on the Service Delivery?. *Analysis of data from focus-group and individual interviews ended up with a causal loop diagram demonstrating potential explanatory mechanisms as shown in Figure 3. Regarding financial incentive as a key performance driver, findings from the qualitative approach revealed many sources of revenues for an EMS unit including regular budget, donation, and user fees apart from those in the two payment systems. Some EMS units under charitable organizations refused to receive payment from the two payment systems altogether since the beginning of those systems. The consequence of public financial audit on a number of local authority agencies had been realized by all key informants working with the agencies and outsiders. Under the current government, news reports had covered a number of top executives of these agencies being dismissed from their offices and they faced financial penalty. Some of our key informants mentioned ending of EMS services under those local authorities. For both EMS units under public and nonpublic agencies, a common perception was unmatched expenditure for provision of EMS services to the amount of revenues from the two schemes.

## 4. Discussion

### 4.1. Progress of the New Payment Systems (Implementation Outcomes)

The findings support shortening of reimbursement time and enabling tracking of data flows. This indicates tangible progress of the implementation of the payment systems. The findings are more remarkable when focusing on the percentage of provinces meeting the NIEMS defined deadline of claim submission. With less demands on data to fulfill the reimbursement processes of direct-pay system as compared to those of E-claim system, it is quite readily understood why progress was more pronounced with direct-pay system than that of E-claim system extra requiring sizable data items to the ITEMS data requirement (Table 3). In fact, missing data as required by the insurance companies had been considered a major obstacle to improve coverage of loss compensation since the early years of implementation of the Road Traffic Accident Victim Compensation Act [11]. The remaining large deficit in the coverage implied by the maximum loss ratio (35%) in the aforementioned (settings and policy interventions) might indicate stagnation in any attempts to fill the gaps. Finally, increasing trends were observed for the number of patients being compensated (93,570,156 cases) and the amount of cash flow from insurance companies to NIEMS for prehospital care (USD 1229;8230;16,663) during the study period according to NIEMS in-house data analysis. However, the increased number of patients could be considered trivial compared to over 300,000 road-traffic injury victims with ambulance transport during the same period [6]. In exchange for those amounts of money NIEMS had to spend, unilaterally, at least USD 5,714 per annum for linking electronic databases of NIEMS and RVP.

### 4.2. Effects on Patient Outcomes

When we turned to explore the effects on patient outcomes of direct-pay and E-claim systems, the negative findings from analysis of huge amount of ITEMS data corresponded with findings from qualitative approaches which indicated probably nonsubstantial amount of financial support from these two sources and probably remarkable gaps in aligning any financial resources to human resource management among prehospital care agencies. A costing study in 2015 on prehospital care services clearly depicted sizable deficits of the current amount of payment to prehospital care units varying from 2.9- to 12.2-fold for BLS level and first responder level, respectively. In effect, several key informants from all the study sites clearly mentioned repeated attempts to feed back on the financial and human resource issues to NIEMS over the past several years. Furthermore, plausible explanations for some EMS units under charitable organizations which refused to receive payment from the two payment systems altogether included (a) hidden concern about being subjected to public financial audit and (b) a perception of disproportionally low contribution of revenues from the two systems to justify the risk of subjection to the public financial audit.

### 4.3. Leadership and Governance Functions

We believe that to a certain extent NIEMS did respond to the bottom-up feedback by sponsoring the study on the cost of prehospital care services as mentioned above. Subsequent to the costing study, NIEMS took the direct-pay initiative to improve the process of previous indirect-pay reimbursement system. Furthermore, NIEMS has tried to mobilise more financial resources by collaborating with RVP on the E-claim system. Despite limited progress in the financial reform initiatives as evident in our study, at least these concrete steps undertaken by NIEMS suggested an inherent responsiveness property of this national lead agency for advancing prehospital care services in Thailand. In this report, we used the term “responsiveness” differently from that in some earlier articles such as Calvello et al. which considered “responsiveness” as responding to patient needs [12]. Yet the essence of the meaning is quite the same, i.e., to reflect on leadership and governance functions of prehospital care systems using financial arrangement as an entry point then linked to patient outcomes (Table 6).

### 4.4. Lessons Learnt

Prehospital care is a labor-intensive service which means human resource management (such as training, EMS staff compensation, and welfare) is crucial to ensure good quality of care as depicted in Figure 3. Failure to demonstrate practical improvement in quality of care as a result of the financial reform (Table 6) indicates further need to keep good alignment between financial mechanisms and human resource management. Finally, considering patient outcome as an ultimate goal of prehospital care, there is a need to take into account linkage between financial reform and continuously updating information system to keep track of changes in patient outcome.

## 5. Conclusions

This study might be able to fill the knowledge gaps of applying a health systems framework to shape the development of prehospital care systems in LMIC settings which were considered a priority research area in this field. It is evident that progress has been made in terms of mobilising more financial inputs and improving financial information flow. However, there is no evidence of any changes in patient outcomes and quality of care.

## 6. Limitations

Making use of administrative data in our study, we faced a large number of missing patient outcome data of up to 40%. We believe that this might result in underestimation of undesirable outcomes. Nonetheless given similar percentages of the missing data among comparison groups, it is unlikely to affect our estimation of the associations shown in Table 6. This pointed out the importance of strengthening information systems in order to improve leadership and governance functions of the prehospital care systems. Employing multiple sources of qualitative data to cross-validate the findings from each specific source enabled us to minimize potential biases associated with a single source.

## Figures and Tables

**Table:** (a) Population and EMS characteristics of selected provinces

Type of EMS unit	Selected provinces								Whole country
	KK^1^	UB^1^	CR^1^	CB^1^	UT^2^	NP^2^	NG^2^	PK^2^	
ALS	24	20	2	3	9	12	4	7	555
BLS	17	27	3	3	4	83	6	1	657
FR	147	71	97	66	26	15	30	11	4669
Non-registered unit	79	159	67	29	59	38	27	8	3971
Population density	162.4	115.2	102.6	81.2	59	396.7	60.7	635.5	128.1
Number of population	1,767,601	1,813,088	1,198,218	514,616	462,618	860,246	253,112	345,067	65,729,098

^1^ Provinces with EMS agencies participating in direct-pay system.

^2^ Provinces without EMS agencies participating in direct-pay system.

**Table:** (b) Participant characteristics for selected provinces in focus-group or individual interview sessions

Type of EMS unit	Selected provinces								Total
	KK^1^	UB^1^	CR^1^	CB^1^	UT^2^	NP^2^	NG^2^	PK^2^	
Provincial health office	3	1	2	3	3	3	5	4	24
Administrators									
Referral hospital	3	2	2	3	3	2	0	2	17
Secondary care hospital	0	3	0	3	2	3	8	3	22
Local authority	3	4	2	3	3	2	1	1	19
RVP	2	0	0	1	1	2	1	2	9
EMS units	6	20	5	3	5	3	4	2	48
Total	17	30	11	16	17	15	19	14	139

^1^ Provinces with EMS agencies participating in direct-pay system.

^2^ Provinces without EMS agencies participating in direct-pay system.

**Table 2:** List of themes and subthemes for guiding data gathering in focus-group or individual interview sessions.

Themes	Subthemes
Governance	Structure and functions of provincial Health office
	Functioning of the PHO in relation to NIEMS and other key actors in a province

Service delivery	Structure and functions of EMS agencies, EDC, and hospital emergency departments
	Scope and volume of the services

Human resources	Staff mix, number of staffs, turn-over rate
	Qualification and certification; capacity building
	Compensation, welfare

Financing	Sources of revenues; cash flows; financial status
	Direct experiences with the two systems
	Reflections on principles of the two systems

Information	Structure and functions of information systems
	Utilization of data and information to support functioning of other building blocks in EMS systems

Technology	Logistics for the delivery of EMS services: timeliness, relevance, adequacy

**Table 3:** Time flow of claim data under direct-pay and e-claim systems adjusted by project-year of participating.

Operation description	Average time taken					
	Direct-pay system			E-claim system		
	r^1^	(95% CI)	p-value	r^1^	(95% CI)	p-value
Call taking to complete submission of financial claims (days)	-10.47	(-30.79, 9.06)	0.294	-8.07	(-27.34, 11.18)	0.411
Call taking to initial submission by EDC (hours)	-62.02	(-173.27, 49.22)	0.275	-45.42	(-155.36, 64.51)	0.418
Complete submission by EDC (days)	-5.11	(-20.51, 10.73)	0.527	-3.04	(-18.63, 12.54)	0.702
Complete submission by EMS units (days)	-7.31	(-24.77, 9.99)	0.407	-7.94	(-24.99, 9.11)	0.361
Percentages of financial claim being made in time (%)	18.18	(4.58, 32.08)	0.01	9.69	(-4.49, 23.89)	0.181

r^1^ = coefficient adjusted by projects and year taking into account intraprovincial variation.

**Table 4:** Time flow of claim data under double system compared with single system adjusted by project-year of participating.

Operation description	Average time taken								
	Direct pay + E-claim system			Direct-pay system only			E-claim system only		
	r^1^	(95% CI)	p-value	r^1^	(95% CI)	p-value	r^1^	(95% CI)	p-value
Call taking to complete submission of financial claims (days)	-16.91	(-42.87, 9.06)	0.202	-11.88	(-37.11, 13.35)	0.356	-9.36	(-41.75, 23.03)	0.571
Call taking to initial submission by EDC (hours)	6.00	(-3.24, 15.24)	0.203	2.00	(-6.98, 10.98)	0.662	-2.18	(-13.71, 9.36)	0.712
Complete submission by EDC (days)	-9.28	(-30.33, 11.78)	0.388	-8.24	(-28.7, 12.22)	0.430	-6.44	(-32.7, 19.83)	0.631
Complete submission by EMS units (days)	-17.02	(-39.86, 5.82)	0.144	-12.43	(-34.63, 9.76)	0.272	-13.77	(-42.26, 14.73)	0.344
Percentages of financial claim completed in time (%)	23.49	(5.11, 41.85)	0.012	16.18	(-1.67, 34.02)	0.076	5.28	(-17.64, 28.19)	0.652

r^1^ = coefficient adjusted by projects and year taking into account intraprovincial variation.

**Table 5:** Themes and representative quotations related to how the direct-pay system and e-claim system initiative might influence overall performance of EMS systems.

Themes	Representative quotations
Governance	“Previously, we were burdened by auditing and data-entry functions of claim papers pooled from all EMS units. Under the direct-pay system, we no longer do the data entry and the money transfer to EMS units.” (staff of provincial health office in a province with participating EMS units)

Service delivery	“It is very difficult to cover remote rural areas with EMS services due to poor road conditions and low population density.”(staff of provincial health office in a province with or without participating EMS units)

Human resources	“We worried about EMS practitioners working for local authorities or charitable organizations have been under compensated given the amount of minimum wage declared by the government…. turnover rate of these folks is usually high.”(administrative staff of a local authority)

Financing	“These days (under direct-pay system), we do not need to wait for EDC to fill ITEMS data form for each patient in order to fill in our part on the same data form.” (EMS practitioner)

Information	“We can trace whether the number of claims we made was accordingly submitted to NIEMS for reimbursement…this was not possible in the past.” (hospital administrator)
	“E-claim system automatically filled in the data related to the license plate number of a car involved in a crash accident such as an insurance policy number.” (hospital administrator)

Technology	“RVP provided an application on mobile phone to facilitate transferring of on-scene capturing of license plate numbers or chassis numbers of motor vehicles involved in a crash event.”(EMS practitioner)

**Table 6:** Comparison of quality of care processes and patient outcomes between participating EMS units and nonparticipating EMS units under the direct-pay system and the E-claim system.

Quality indicator	Direct-pay systems					E-claim system				
	Non-participating unit		Participating unit		*P*	Non-participating unit		Participating unit		*P*
	n	(%)	n	(%)		n	(%)	n	(%)	
Response time < 10 minutes	964344		2241117			374402		442976		
Yes	821646	(85.2)	1896709	(84.6)	<0.001	333783	(89.2)	387860	(87.6)	<0.001
No	142698	(14.8)	344408	(15.4)		40619	(10.8)	55116	(12.4)	

Airway management	320224		699907			89358		114152		
Inappropriate	1574	(0.5)	2217	(0.3)	<0.001	314	(0.4)	394	(0.3)	<0.001
Appropriate	305193	(95.3)	665803	(95.1)		83872	(93.9)	109306	(95.8)	
Did not do	13457	(4.2)	31887	(4.6)		5172	(5.8)	4452	(3.9)	

Bleeding control	196361		367564			181161		224923		
Inappropriate	569	(0.3)	1183	(0.3)	<0.001	572	(0.3)	572	(0.3)	<0.001
Appropriate	186672	(95.1)	342220	(93.1)		171955	(94.9)	216896	(96.4)	
Did not do	9120	(4.6)	24161	(6.6)		8634	(4.8)	7455	(3.3)	

Fluid replacement	62235		148658			21392		23348		
Inappropriate	200	(0.3)	480	(0.3)	<0.001	58	(0.3)	62	(0.3)	0.500
Appropriate	59199	(95.1)	143634	(96.6)		20392	(95.3)	22204	(95.1)	
Did not do	2836	(4.6)	4544	(3.1)		942	(4.4)	1082	(4.6)	

Fracture stabilization	134012		265740			123190		158050		
Inappropriate	834	(0.6)	1683	(0.6)	<0.001	797	(0.6)	945	(0.6)	<0.001
Appropriate	123614	(92.2)	241438	(90.9)		115010	(93.4)	149619	(94.7)	
Did not do	9564	(7.1)	22619	(8.5)		7383	(6)	7486	(4.7)	

Outcome of treatment	1212785		2556614			449156		508811		
Death	15779	(1.3)	24767	(1)	<0.001	4410	(1)	5110	(1)	<0.001
Referral or length of stay > 30 days	103682	(8.5)	218784	(8.6)		41751	(9.3)	51939	(10.2)	
Length of stay < 30 days	642944	(53)	1351751	(52.9)		216169	(48.1)	242023	(47.6)	
Unknown result	17644	(1.5)	22457	(0.9)		5268	(1.2)	5522	(1.1)	
N/A	432736	(35.7)	938855	(36.7)		181558	(40.4)	204217	(40.1)	

## Data Availability

Datasets used in this report may be requested from the authors.
